# Cloning, characterization, and expression analysis of the pig (*Sus scrofa*) C1q tumor necrosis factor-related protein-5 gene

**Published:** 2012-01-17

**Authors:** Jeffrey R. Sommer, Venkata R.M. Chavali, Sean G. Simpson, Radha Ayyagari, Robert M. Petters

**Affiliations:** 1 Department of Animal Science, North Carolina State University, Raleigh, NC; 2 Department of Ophthalmology, University of California, San Diego, La Jolla, CA

## Abstract

**Purpose:**

Autosomal dominant early-onset long anterior zonules (LAZs) and late-onset retinal degeneration (L-ORD) in humans are associated with the S163R mutation of the complement 1q-tumor necrosis factor related protein-5 (*CTRP5*) gene. For using the pig as an L-ORD model for the study of pathology, we cloned, characterized, and studied the expression profile of pig *CTRP5* (p*CTRP5*).

**Methods:**

The p*CTRP5* was cloned and sequenced from porcine genomic DNA. Bioinformatic analysis was done to evaluate the functional domains present in the p*CTRP5* using PROSITE tools. The V5 epitope-tagged constructs of p*CTRP5* and the mammalian promoters, elongation factor 1-α (EF) promoter and 579 bp of the putative promoter located upstream to p*CTRP5* DNA, were used for in vitro expression analysis. The p*CTRP5* expression, protein size, and cellular localization were studied in transiently transfected Cos-7 or ARPE-19 cells by western blot analysis using anti-CTRP5 and anti-V5 epitope antibodies. Expression of p*CTRP5* in the pig eye tissues was analyzed by western blot analysis, real-time PCR, and immunohistochemistry.

**Results:**

As predicted, p*CTRP5* showed a 92% DNA homology and 98% amino acid homology with human *CTRP5* (h*CTRP5*). Bioinformatic analysis revealed the presence of an alternate in-frame translational start site upstream to the presumed initiator codon. The presence of a putative promoter region upstream to the p*CTRP5* was identified. The putative p*CTRP5* promoter was found to be functional by western blot analysis. The size of the p*CTRP5* protein (pCTRP5) was consistent with its predicted molecular weight, indicating that the potential alternative start site was not used. Western blot and RT–PCR analyses showed that pCTRP5 was predominantly expressed in RPE, a pattern of expression consistent with that found in mouse and human eyes.

**Conclusions:**

The sequence and genomic organization of p*CTRP5* was found to be similar to the human homolog. The DNA and protein sequence of p*CTRP5* are highly homologous to h*CTRP5*, indicating that they are highly conserved. A putative promoter region (579 bp) present upstream to p*CTRP5* was found to be functional and was able to drive the expression of the p*CTRP5* gene cloned downstream. The tissue distribution in the eye and the expression profile of pCTRP5 in transiently transfected cells is consistent with hCTRP5 expression. Immunohistochemistry analysis of the pig retinal sections revealed localization of pCTRP5 to the apical and basolateral regions on the RPE and in the ciliary body. The potential in-frame alternate start site was found to be nonfunctional by western blot analysis of transiently transfected cells. Similarities between human and pig *CTRP5* and the presence of an area centralis region in the pig similar to the human macula, together with its large eyeball size, makes the domestic pig a good model for the study of LAZs and L-ORD.

## Introduction

Late onset retinal macular degeneration (L-ORD) is an autosomal dominant retinal degeneration that is characterized by bilateral vision loss, dark-adaptation abnormalities, drusenoid deposits, lens anterior zonules, retinal degeneration, and choroidal neovascularization in humans [1-3]. Patients with L-ORD often present symptoms indistinguishable from early-stage age-related macular degeneration (AMD) or from retinal degeneration (RD) in its later stages. However, anterior segment abnormalities with long anterior zonules (LAZs) are seen only in L-ORD patients and are not observed in AMD or RD. L-ORD is known to be caused by a single missense mutation, S163R, in a conserved domain of the C1q tumor necrosis factor-related protein 5 gene (*CTRP5*) [3]. Patients with the S163R heterozygous mutation showed abnormal lens zonules (in the second decade), dark adaptation abnormalities in the fourth decade, progressing to drusen and macular degeneration with choroidal neovascularization (fifth decade). Some of the patients developed iris atrophy and secondary glaucoma in their 60s [3-5].

*CTRP5* is a short chain collagen gene that encodes a 25 kDa secretory glycoprotein with three conserved domains: a signal peptide (residues 1–15), a collagen domain (residues 30–98) containing 23 uninterrupted gly-X-Y repeats, and a C1q domain (residues 99–243). It is highly expressed in the RPE and ciliary epithelial layers in the eye [6]. Expression of CTRP5 is also reported in serum, adipocytes, and other tissues in the body [7,8]. The protein and DNA sequence of *CTRP5* is highly conserved in mammals, birds, and zebra fish [9]. *CTRP5* is known to express as a dicistronic transcript and is located in the 3′ untranslated region of the membrane-type frizzled-related protein (*MFRP*) gene [10]. A functional promoter for human *CTRP5* (*hCTRP5*) has been identified in its 5′ region [11].

**Figure 1:**
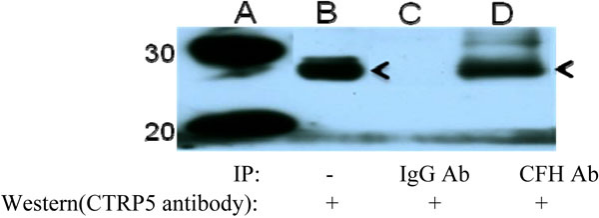
Western blot indicating an interaction between CTRP5 and CFH. The blot was probed with an hCTRP5 protein antibody. Lane A, with molecular weight markers from the same blot, is presented adjacent to lanes B-D: lane B is purified V5 tagged hCTRP5 protein; lane C is the immunoprecipitated (IP) fraction with the IgG control antibody and the western blot with the hCTRP5 antibody; lane D is the IP fraction with 1.5 μg human complement factor H antibody and western blot with hCTRP5 antibody.

The CTRP5 protein has been shown to interact with complement factor H (CFH), which has been reported to be a major genetic factor associated with AMD and an early-onset recessive drusen phenotype (Figure 1) [12]. Apart from CFH, CTRP5 is also known to interact with membrane-type frizzled-related protein (MFRP) [13]. The C1q domain present in *CTRP5* is known to strongly interact with the complement C1r/C1s, Uegf, Bmp1 (CUB) domains joined by LDLa (together known as the CUBT domain) in *MFRP*. Mutations in *MFRP* are reported to cause an autosomal recessive syndrome of nanophthalmos, retinitis pigmentosa, foveoschisis, and optic disc drusen in human subjects and retinal degeneration in the *rd6* mouse model [10,14]. The highest levels of *CTRP5* and *MFRP* expression were detected in RPE and the ciliary body, the tissues that are involved in the disease pathology [3]. Even though CTRP5 is known to interact with CFH and MFRP, its function in disease pathology is not known to date.

There are many currently existing mouse models for studying AMD that mimic most of the phenotypic features present in humans [15]. However, the greatest phenotypic difference between humans and mice relates to ocular size. In addition, to understand the mechanism underlying L-ORD or AMD, studying an animal model having a foveal pit or macula is justified, rather than studying mouse models, since mice lack both these features. Since the pig has a distinct macula-like region, the area centralis, with a high concentration of cones [16-18], it will be a better model for studying macular degeneration. Understanding the structure and expression of *CTRP5* in the pig will enable us to manipulate it and to make models to mimic the disease in human L-ORD patients. In the present study, we describe the cloning, sequencing, and characterization of *CTRP5* in pigs (p*CTRP5*, in *Sus scrofa*) and its putative promoter. These studies will provide the background for the development of a pig model of LAZs and L-ORD for the investigation of therapeutic interventions.

## Methods

All animal protocols were conducted in accordance with state and federal guidelines and were approved by the Institutional Animal Care and Use Committee, North Carolina State University.

### Cloning p*CTRP5* and constructing the expression vector

PCR primers, CTRP5 6up (5′-GCG ACA CCA CGA GTT ATT TCC CTG-3′) and 2082lp (5′-AGC CCT CCC TTC TGC CTG AAC AC-3′), were used to amplify 2,146 bp of *CTRP5* from the pig genomic DNA using Phusion Hot Start High-fidelity DNA polymerase (New England BioLabs, Ipswich, MA). The PCR amplicon was agarose gel purified and annealed with “A” overhangs before cloning into the mammalian expression vector pEF6/V5-His-TOPO (Invitrogen, Carlsbad, CA). The positive clones containing p*CTRP5* were confirmed by PCR amplification using the nested primer set CTRP up/lp (5′-CCT TCG ACC GCG TGC TG-3′ and 5′-AGC AGA GGC TGG CTT GGG C-3′), which flanks the Ser163 codon in exon 2 of p*CTRP5*. This vector was called pEF CTRP5 wt. An Arg163 mutation was introduced by site-directed mutagenesis into the pEF CTRP5 wt plasmid using the 5′ phosphorylated primers CTRP5 AGG mut F (5′[P]-CTC TTG GAG TCT GGG AGG AGC A-3′) and CTRP5 AGG mut R (5′[P]-CTG GTC AAG AAT GGC GAG TCC AT-3′) for amplification and then recircularization to make the pEF CTRP5 mt plasmid. Restriction endonuclease digestion analysis with NheI was used to screen for the mutation because the C→G mutation eliminates the NheI site. Positive clones containing the mutant CTRP5 were confirmed by sequencing. A possible promoter and alternative in-frame ATG start site found in the 5′ upstream sequence of the pig genomic DNA was removed from with the 5′ phosphorylated primer set: ATG out up (5′[P]-GAG GCC GGG AGC GAG GCT TGT C-3′) and ATG out lp (5′ [P]-ACT GGT GTC GCA AGG GCA ATT C-3). The resulting amplicon was recircularized. We called this version of the CTRP5 “S” for short (pEF CTRP5 S).

For ease of detection of protein expression in transient transfection assays, a 3′ in-frame fusion was made with the V5 epitope of the pEF6/V5-His-TOPO cloning vector. The abbreviation “T” (for “tagged”) was used to denote plasmids that were V5 epitope-tagged (e.g., pEF CTRP5 wt T). To investigate the promoter activity of the CTRP5 upstream sequence, we made constructs with EF promoter and putative pCTRP5 promoter sequences cloned upstream to p*CTRP5*. These constructs were transiently transfected into ARPE-19 cells or Cos-7 cells to study the pCTRP5 (protein) expression by western blot and immunocytochemistry analysis.

### 5′ RACE analysis of p*CTRP5*

To determine the transcription start site of p*CTRP5*, a 5′ RACE was performed using the RNA isolated from pig RPE tissue using an RNeasy Mini isolation kit (Qiagen, Valencia, CA). Ten micrograms of the total RNA was used with the First Choice RLM RACE kit (Invitrogen). The cDNA was synthesized using an Enhanced Avian RT first-strand synthesis kit (Sigma-Aldrich, St. Louis, MO) at 65 °C using a primer specific to the CTRP5 coding sequence (Primer Race1: 5′-CCC TCG CCT TTC TCT CCC-3′). PCR was performed with NEB’s Long Amp DNA polymerase using these primers: 5′ RACE Outer Primer (5′-GCT GAT GGC GAT GAA TGA ACA CTG-3′) and reverse Race2 (5′- GCC TTT CTC TCC CGG AGC-3′). A nested PCR reaction using the same conditions and the following primers was performed: 5′ RACE Inner Primer (5′-CGC GGA TCC GAA CAC TGC GTT TGC TGG CTT TGA TG-3′) and reverse Race6 (5′-GCG TGC CAG GAA GGC CGG-3′). The amplification product was gel-purified and sequenced with the reverse Race6 primer.

### Bioinformatic analysis and prediction of a core promoter region for p*CTRP5*

A bioinformatic search was done with p*CTRP5* DNA and protein sequences using NCBI-BLAST to check for homology with other DNA and proteins in the database. ClustalW software was used to determine the percentage of homology of p*CTRP5* with its orthologs for both DNA and protein. The presence of functional protein domains was tested using PROSITE tools. We used both PROSCAN Ver. 1.7 and TSSG server software to determine the presence of a putative promoter and of transcriptional start sites in the 5′ region of the p*CTRP5* sequence [19]. We also screened the 5′ sequence upstream to p*CTRP5* for the presence of CpG islands using CpG islands searcher software [20].

### Transfection of Cos-7 and ARPE-19 cells

Cells were transiently transfected with the expression constructs using Lipofectamine 2000 reagent (Invitrogen) according to the manufacturer’s instructions [21]. Four hours after transfection was initiated, the transfection mix was replaced with serum-containing medium and incubated for another 48 h before experiments were performed. Mock transfections were performed using Lipofectamine 2000 only [21].

### Analysis of wild-type and mutant pig CTRP5 expressed in Cos-7 cells

Cos-7 cells were transfected with the wild type (p*CTRP5* wt T) and mutant (p*CTRP5* mt T) expression constructs according to previously published protocols [21]. The expression of *hCTRP5* under the control of cytomegalovirus (CMV) promoter [6] was used as a positive control. The cells were harvested after 48 h posttransfection to prepare lysates. A protease inhibitor cocktail (Sigma Aldrich) was added to the lysis buffer (50 mM Tris-HCl [pH 7.4], 0.15 M NaCl, 1 mM EDTA, 0.1% Triton X-100, and 0.1% [W/V] SDS). Western blot analyses were performed with 30 µg reduced proteins (by adding 0.5% β-mercaptoethanol to the SDS sample buffer) separated on 10% Bis-Tris gel (Invitrogen). After the western transfer, the blot was probed with rabbit anti-CTRP5 antibody [6].

### Quantitative expression of pCTRP5 in different eye tissues

Quantitative RT–PCR (qRT–PCR) and data analysis were performed according to previously published protocols [6]. Initial denaturation of the cDNA was done at 94 °C for 1 min for one cycle followed by 40 cycles of denaturation at 95 °C for 10 s, annealing at 56 °C for 30 s, and elongation at 72 °C for 40 s. Primers 5′-GTC CAT CGC CTC TTT CTT CC-3′ (forward primer) and 5′-ATG CCA ATG TAG TCA CCC AC-3′ (reverse primer) were used for amplification of p*CTRP5*. The expression of p*CTRP5* was determined in pig eye tissues (cornea, ciliary body-iris [CB-I], lens, retina, and RPE). In addition, four housekeeping genes—*Gapdh*, *HPRT*, β-actin (*Actb*), and *RpL19*—were also analyzed as controls to normalize the expression of p*CTRP5*. Expression levels (±standard error of the mean [SEM]) were calculated by analyzing at least three independent samples with replicate reactions and presented on an arbitrary scale that represented its expression over the housekeeping gene. The data was presented using the *HPRT* gene for normalization.

### Immunohistochemistry

For immunolocalization of antigens, eyes from 7.4-month-old pigs were fixed in 4% paraformaldehyde in 0.1 M phosphate buffer. Eyes in fixative were thoroughly washed with 1X PBS, cryoprotected in 30% sucrose, and embedded in OCT before sectioning. To localize pCTRP5 in the eye tissue, immunohistochemistry (IHC) was performed using anti-CTRP5 polyclonal antibody, as described earlier [6,11]. Sections were mounted in mounting medium containing DAPI, they were observed with a Zeiss confocal microscope, and images were captured.

## Results

### Characterization of p*CTRP5*

**Figure 2:**
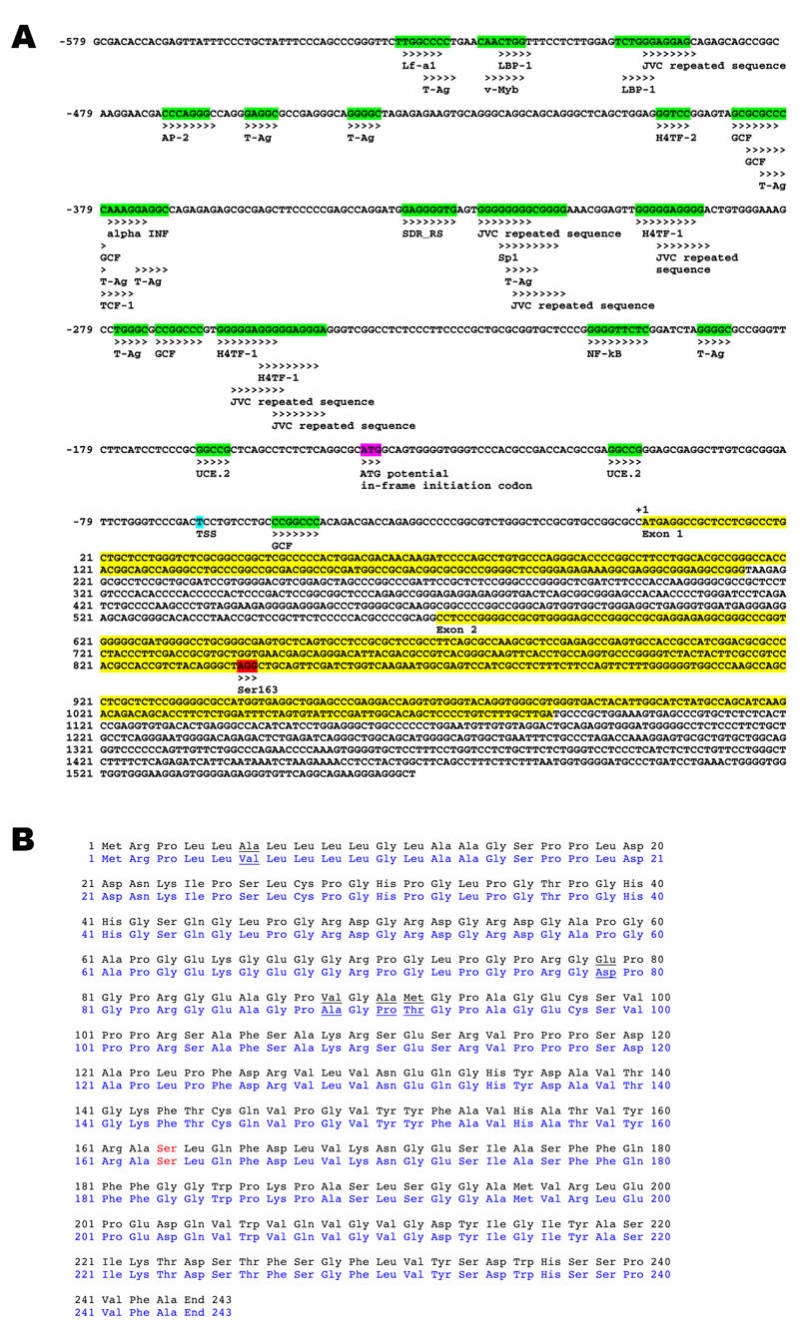
Genomic structure of p*CTRP5* and protein homology of pig CTRP5 with the human protein. **A**: The p*CTRP5* was amplified from genomic DNA by PCR, cloned, and sequenced. The p*CTRP5* consists of two exons; its coding sequence is highlighted in yellow, the Ser163 codon is highlighted in red, a potential in-frame alternative start codon is highlighted in pink, the transcriptional start site (TSS) is highlighted in blue, and various transcription factor binding sites are highlighted in green. **B**: The protein homology between the pCTRP5 and hCTRP5 protein is represented. The pCTRP5 protein sequence is shown in black and the human in blue. The underlined residues indicate the five amino acid differences between the pig and human protein. The Ser shown in red indicates residue 163.

A 2146 bp fragment of pig genomic DNA containing the presumed coding region for *CTRP5* (Figure 2A) was cloned into the mammalian expression vector pEF6/V5-His-TOPO (referred to as pEF CTRP5 wt). Amplification with nested *CTRP5*-specific PCR primers and subsequent sequencing confirmed the presence of the p*CTRP5* coding sequence (Figure 2A).

Sequencing analysis of the cloned p*CTRP5* revealed it is 732 bp long with two exons that might encode for a 243 amino acid protein (molecular weight [MW] was nearly 25.5 kDa). DNA sequence’s homology with its human ortholog is 92% and amino acid homology is 98%; with variations at only 5 amino acid residues (Figure 2B). The sequence comparisons of p*CTRP5* with other vertebrate and invertebrate sequences showed a high conservation of the C1q domain, which is the functionally active domain of CTRP5 [7,22]. PROSITE analysis showed the presence of the collagen domain to be from 36 to 95 amino acids and of the C1q domain to be from 105 to 232 amino acids in pCTRP5 protein. The region between 102 and 235 amino acids showed homology with the tumor necrosis factor-like protein family of proteins. Analysis of the genomic sequence indicated that a potential in-frame alternative start codon 141 bp upstream of the predicted p*CTRP5* start codon, if translated, would result in an extra 47 amino acids at the N-terminus, to yield a protein of the predicted size of nearly 31 kDa (Figure 2A).

The CpG island search software detected the presence of a single CpG island of 578bp with a GC content >70.6%. The observed CpG/expected CpG ratio in this region is greater than 0.65. Presence of a CpG island in the putative promoter region of pCTRP5 may help either to initiate DNA replication or transcription.

### Transient expression of CTRP5 in Cos-7 and ARPE-19 cells

**Figure 3:**
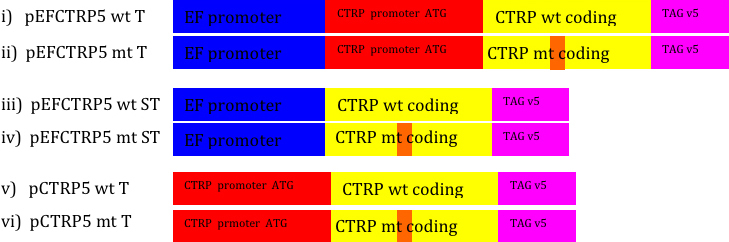
Expression constructs designed for studying expression of p*CTRP5* and p*CTRP5* promoter activity. Six constructs were made to investigate p*CTRP5* expression and putative pCTRP5 promoter activity. The initial vector was pEF CTRP5 wt. All other vectors were based on modifications to this expression vector. Figure abbreviations are as follows: wt=wild type Ser163 allele, mt=Arg163 mutation, T=V5 epitope tagged, S=p*CTRP5* short. The short p*CTRP5* genomic fragment is missing 461 bp of the upstream sequence, including the possible promoter and alternative in-frame start site. Constructs made with EF1α promoter were used as experimental controls for promoter expression studies.

To test whether the cloned p*CTRP5* was capable of expressing pCTRP5, we cloned 2164 bp of p*CTRP5* downstream to the human elongation factor 1α (EF) promoter (pEF CTRP5 wt). We used this vector to produce a series of expression vectors, including V5 epitope “tagged” (T) CTRP5 and “short” (S) versions that did not contain a 5′ upstream genomic sequence that included a putative p*CTRP5* promoter and a potential alternative translational start site (pCTRP5 wt T and pCTRP5 wt S) expression vector. R163 mutant (mt) variations of each plasmid were also produced to test for their expression (Figure 3). The resulting plasmids were transiently transfected into Cos-7 cells and ARPE-19 cells independently, and the expression of pCTRP5 protein was studied using western blot analysis.

**Figure 4:**
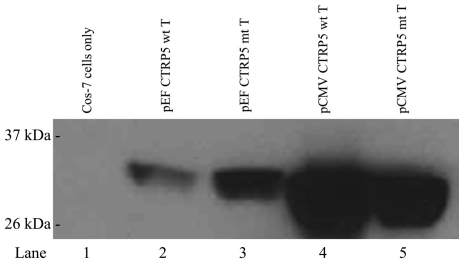
Western blot of lysates of cells transfected with pig *CTRP5* and human *CTRP5* constructs. Western blot analysis was performed with anti-V5 antibody on lysates from Cos-7 cells transfected with pEF CTRP5 wt T (lane 2), pEF CTRP5 mt T (lane 3), pCMV CTRP5 wt T (lane 4), pCMV CTRP5 mt T (lane 5), and nontransfected Cos7 cells (lane 1). Lanes 4 and 5 were transfected with hCTRP5 expression vectors as a positive control. Detection with anti-V5 antibody produced a single band with a molecular weight around 31 kDa for both the pCTRP5 (lanes 2 and 3) and hCTRP5 (lanes 4 and 5) V5-tagged protein.

Western blot analysis with Cos-7 cells transfected with the plasmids pEF CTRP5 wt T and pEF CTRP5 mt T were incubated with an anti-V5 antibody to detect the CTRP5 fusion protein (Figure 4). The expression of the pCTRP5 V5 fusion protein indicated the functionality of the cloned p*CTRP5* in producing CTRP5 (~31 kDa). The expression of pCTRP5 was comparable to the levels of CTRP5 produced by the V5 epitope-tagged h*CTRP5* gene expressed under the control of the CMV promoters (pCMV CTRP5 wt T and pCMV CTRP5 mt T) that were used as controls.

### Promoter analysis of p*CTRP5*

Promoter identification software detected a TATA-less putative promoter region of an approximately 579 bp sequence upstream of the predicted p*CTRP5* initiation codon. The Proscan Version 1.7 software tool predicted this promoter region to be in proper orientation to the start site, with a promoter score of 53.45 (promoter cutoff=53.0). The TSSG program, which searches for human PolII promoter regions, predicted a promoter with a transcriptional start site at −245 bp from the start of translation, with a linear discriminant factor (LDF) score of 7.59 (threshold for LDF=4.00). Analysis of the putative p*CTRP5* promoter region (579 bp) also identified several possible transcription-factor binding sites, including one site of NF-kB and four sites for the Sp1 binding site within the sequence (Table 1). The presence of a TATA box or GATA box was not detected, indicating that the p*CTRP5* promoter might be a TATA-less promoter.

**Table 1:** List of transcription factors binding sites identified in the 579 bp upstream promoter region of the pig *CTRP5* gene.

**Binding site**	Identification*	**Location from start of translation (bp)**
Alpha INF	S01153	(−378 to −373)
AP-2	S01936	(−470 to −463)
GCF	S01964	(−54 to −48) (−169 to −163) (−271 to −265) (−385 to −379) (−387 to −381)
H4TF-1	S01969	(−256 to −248) (−262 to −254) (−304 to −293)
H4TF-2	S00742	(−398 to −394)
JVC repeat sequences	S01193	(−254 to −247) (−260 to −253) (−299 to −292) (−319 to −312) (−324 to −317) (−500 to −493)
LBP-1	S02121, S00487	(−503 to −499) (−521 to −517)
Lf-A1	S00250	(−534 to −529)
NF-kB	S01498	(−208 to −200)
SDR	S01561	(−335 to −328)
Sp1	S00781, S00978 S00857, S00979	(−321 to −313)
T-Ag	S00973, S00974 S02135, S01375	(−192 to −188) (−277 to −273) (−320 to −316) (−374 to −370) (−443 to −439) (−458 to −454)
TCF-1	S02023	(−379 to −375)
UCE.2	S00437	(−165 to −161) (−105 to −101)
v-Myb	S01896	(−523 to −518)

Selected transcription factor binding sites were identified using Proscan V1.7 and TSSG searches. *Motif identificators from the Ghosh database.

**Figure 5:**
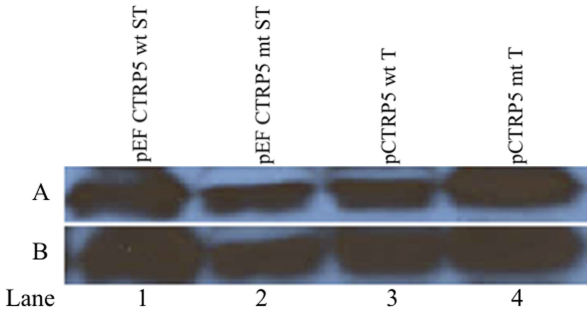
Western blot analysis of Cos-7 cells lysates transfected with pig CTRP5 promoter constructs. **A**: Cos-7 cells were transfected with pEF-CTRP5 wt ST (lane 1), pEF-CTRP5 mt ST (lane 2), pCTRP5 wt T (lane 3), and pCTRP5 mt T (lane 4) and the cell lysates were incubated with anti-V5 antibody. **B**: The membrane was stripped and incubated with anti-CTRP5 polyclonal antibody. A single protein with as size of approximately 31 kDa was identified using either antibodies indicating the V5 tagged CTRP5 protein is being expressed (**A** and **B**). The putative pCTRP5 promoter is capable of expressing the V5 tagged protein confirming the functionality of the pCTRP5 promoter (**A** lanes 3 and 4; **B** lanes 3 and 4).

To test the functionality of the predicted promoter, the putative promoter region (579 bp) was cloned upstream to the start site of p*CTRP5* and analyzed for its ability to express pCTRP5. Immunocytochemistry analysis was done to analyze protein expression in ARPE-19 cells transfected with the pCTRP5 wt T and pCTRP mt T constructs. The 579 bp p*CTRP5* promoter was capable of expressing pCTRP5, which was detected using anti-CTRP5 antibody and anti-V5 tag antibodies (data not shown). Furthermore, western blot analysis of Cos-7 transfected with p*CTRP5* under the control of the 579 bp p*CTRP5* promoter demonstrated high levels of expression of both the wild-type and mutant pCTRP5 proteins (Figure 5). The above results confirmed the functionality of the putative pig promoter. The pEFα promoter driving expression of p*CTRP5* V5 fusion genes were used as controls.

### Determination of the translational start site of p*CTRP5*

**Figure 6:**
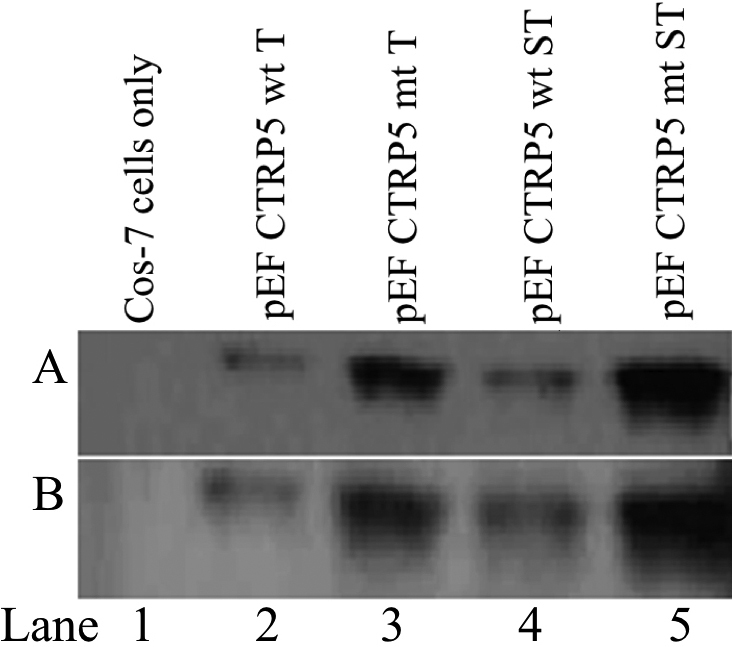
Western blot analysis for the detection of functional ATG start site for pCTRP5. **A**: western blot analysis of Cos-7 cell lysates transfected with pEF-CTRP5 wt T (lane 2), pEF CTRP5 mt T (lane 3), pEF CTRP5 wt ST (lane 4), pEF CTRP5 mt ST (lane 5), and nontransfected Cos-7 cells (lane 1) incubated with anti-CTRP5 polyclonal antibody. **B**: The blot was stripped and incubated with anti-V5 antibody. A single 31 kDa protein was detected using either antibody. This indicates that only the predicted ATG is the functional for the translation of CTRP5 **(A** and **B**).

To investigate the potential translational start site of the p*CTRP5* gene, Cos7 cells were transfected with two different expression constructs. These constructs contained either the potential alternative in-frame start sites (pEF CTRP5 wt T and pEF CTRP5 mt T) or truncated versions of the expression vectors (pEF CTRP5 wt ST and pEF CTRP5 mt ST) that lacked the potential alternative start site (Figure 6). The transfected Cos-7 cell lysates were analyzed using western blot analysis with an anti-CTRP5 antibody and an anti-V5 epitope antibody. Detection with the above antibodies revealed that transfected cells expressed pCTRP5 protein with a molecular weight of 31 kDa. Translation from the in-frame alternative start site would have resulted in the expression of a 36 kDa protein. These results suggest that, even though a potential in-frame ATG was present in exon 1, only the second ATG was used for translation of CTRP5 (Figure 6).

### Determination of the p*CTRP5* transcriptional start site

5′ RACE was performed to determine the transcriptional start site of the endogenous pCTRP5 gene. RNA purified from the pig retinal-pigmented epithelium was used as the starting template for the RACE adaptor ligation and reverse transcription reaction. Sequencing of a PCR product from the 5′ RACE reaction determined that the transcriptional start site was located at −65 bp from the initiation codon ATG (Figure 2A). The detected transcriptional start site (TSS) was found to be downstream of a potential in-frame initiation codon (77 bp downstream to the alternate initiation codon), indicating that it was not used for the generation of the 5′ RACE product.

### Expression of p*CTRP5* in various eye tissues and localization in the eye

**Figure 7:**
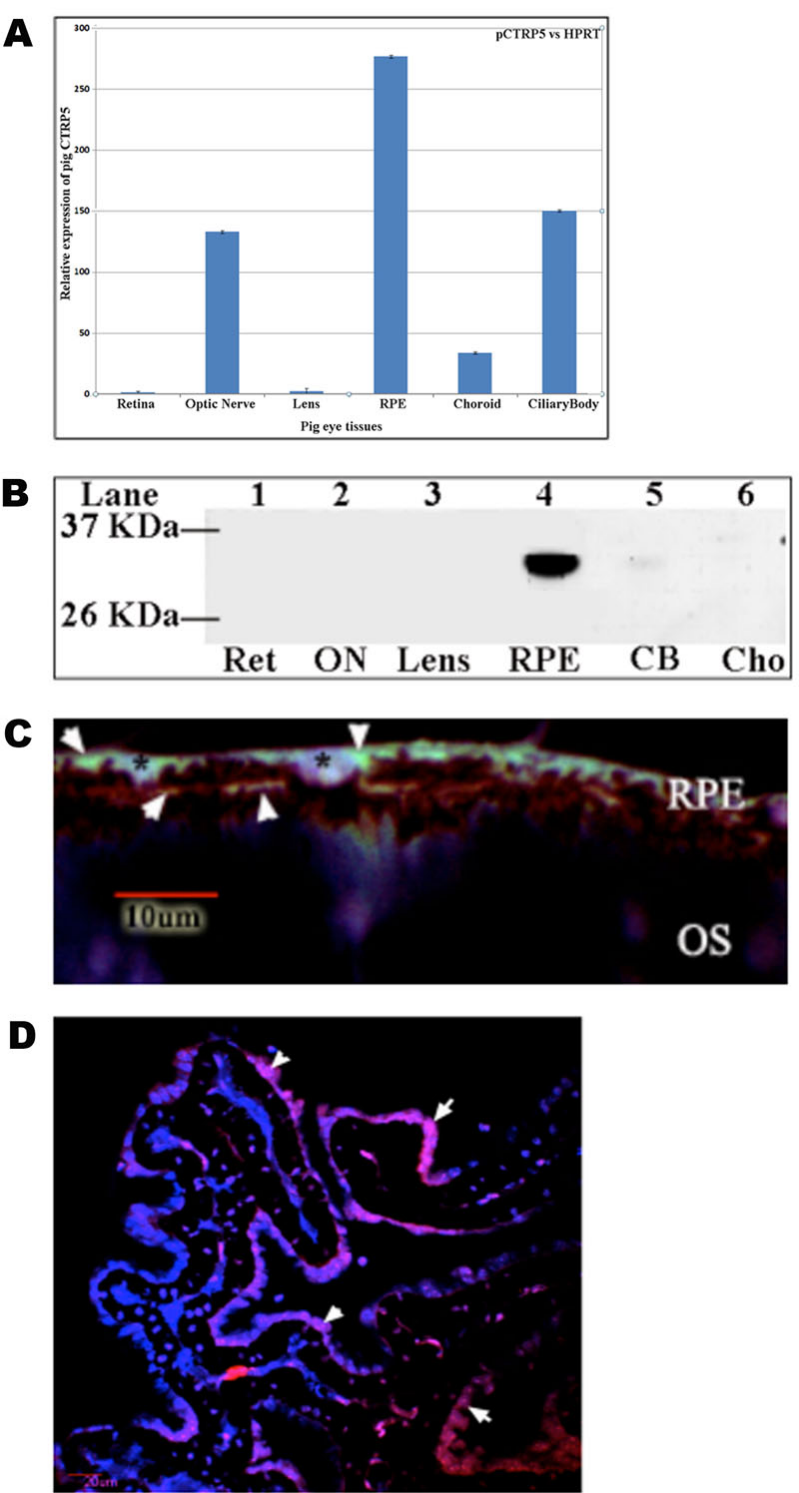
Expression and localization of *CTRP5* in the pig eye. **A**: Expression of p*CTRP5* was studied by qRT–PCR using total mRNA extracted from different tissues of a 222-day-old pig eye. The p*CTRP5* expression is presented as bars using an arbitrary scale on the *y* axis. Values are presented as mean (±SEM) of three independent observations after normalization with the control gene (HGPRT) **B**: western blot analysis of CTRP5 protein extracted from a 222 days pig. Retina (lane 1), optic nerve (lane 2), lens (lane 3), RPE (lane 4), ciliary body (lane 5), Choroid (lane 6). Detection with an anti-CTRP5 antibody shows significant expression of pCTRP5 protein in the RPE with an expected size molecular weight of approximately 31 kDa. **C**: Localization of CTRP5 in retinal sections as evaluated by IHC analysis of retinal sections with human monoclonal anti-CTRP5 antibody and Alexa Fluor 488 staining (green, arrows), nuclei (*) stained with DAPI (blue). **D**: Localization of CTRP5 in the ciliary body as shown by IHC with human monoclonal anti-CTRP5 antibody and Alexa Fluor 555 (red, arrows), nuclei stained with DAPI (blue).

We examined p*CTRP5* gene expression in different pig eye tissues using quantitative reverse transcription polymerase chain reaction (qRT–PCR) on cDNA produced from total mRNA isolated from the retina, optic nerve, lens, RPE, choroid, and ciliary body of a 222-day-old pig. The highest level of p*CTRP5* expression was detected in the RPE, although p*CTRP5* messages were expressed in the ciliary body, optic nerve, and choroid. Minimal expression levels of the p*CTRP5* transcript were detected in the pig retina and lens (Figure 7A).

Western blot analysis was performed using the protein lysates of the above tissues. The pCTRP5 protein was primarily detected within the RPE (Figure 7B) as ~31 kDa protein (Figure 7B) but not in other eye tissues, indicating that the RPE was the predominant tissue expressing the CTRP5 protein. To localize the presence of CTRP5 protein in the pig eye, IHC was performed with an anti-CTRP5 antibody. The pCTRP5 protein was localized predominantly to the basal RPE layers of the eye (Figure 7C). Staining of CTRP5 was also observed on the apical and lateral regions throughout the retina (Figure 7C). From the above analysis it appears that, although the p*CTRP5* transcript was detected in various eye tissues, it was predominantly expressed and localized to the RPE layer in pig eye.

The localization pattern of pCTRP5 in the ciliary epithelium (CE) of pigs was demonstrated by IHC using the anti-CTRP5 antibody. Immunofluorescence microscopy showed that pCTRP5 was localized to predominantly to the apical and few basal membrane surfaces of the pigmented and nonpigmented cells, and was abundantly present in the CE layers (arrows in Figure 7D). Among the ciliary epithelial layers, pCTRP5 was found to be significantly localized in the outer layer between both layers, unlike the mouse CTRP5 that was reported to be localized in the apposed apical membrane surfaces of the CE layers [6]. The variation observed in the localization pattern of CTRP5 between mouse, human, and pig retinal sections could be due to the difference in the specificity of anti-human/-mouse CTRP5 polyclonal antibodies against the pig protein. Similar to the pattern observed in the mouse RPE cells, pCTRP5 also showed a predominantly punctate distribution in the ciliary epithelium [6].

## Discussion

The Ser163Arg mutation in h*CTRP5* is known to cause the autosomal dominant pathology associated with early-onset LAZs and late-onset retinal degeneration [3,9]. Developing suitable animal models that mimic the human phenotype is critical to understanding the disease pathology caused by gene mutations. The phenotype of these patients resembles that of patients with AMD and RD. There are multiple mouse models showing pathological features mimicking those of AMD [15]. In spite of having many phenotypic similarities with AMD patients, the mouse eye differs significantly from the human eye, due to the mouse eye’s smaller size and the absence of a cone-rich region, the macula. A pig model can serve as an alternative best model to study AMD due to the presence of the cone-rich region, called the area centralis, and its bigger eye size compared to that of the mouse. To generate a pig model for L-ORD in future, we cloned and characterized the genomic p*CTRP5* gene. This is the first study describing p*CTRP5* and its expression and localization pattern in the pig eye. This study also confirmed the presence of an independent promoter for p*CTRP5* that is similar to but smaller than the human promoter [11].

To characterize p*CTRP5*, we cloned the gene from genomic DNA and sequenced it. The first release (April 2009) of the high-coverage *Sus scrofa* assembly for chromosomes 1 to 18 and X of the pig genome is available through Ensembl browser [23]. We compared the p*CTRP5* gene sequence we generated with the public domain sequence and found a 100% match between the two sequences. Initally, the *hCTRP5* transcript was found to be present in the 3′ untranslated region of the full length *hMFRP* transcript [6]. We have since identified an autonomous *hCTRP5* promoter [11]. The genomic organization between the coding exons in *pCTRP5* (exon 1 and exon 2) and *hCTRP5* (exon 1 and exon 2) is very similar. They both have the same-size exons of 215 bp and 517 bp for, respectively, the first and second coding exons. The gene sequence homology of both the DNA and protein was compared with other ortholog sequences. The DNA and protein alignments of the p*CTRP5* gene showed, respectively, a 92% DNA homology and a 98% amino acid homology with h*CTRP5*. ClustalW sequence analysis of the 579 bp sequence upstream to the *CTRP5* gene in pigs and humans revealed an alignment score of 74%, indicating that they are conserved.

The *CTRP5* message is reported to be expressed as two isoforms in humans: a dicistronic transcript of *CTRP5* with the *MFRP* gene in its 5′ region, and a monocistronic transcript without *MFRP* [24]. The CUB domains present in human *MFRP* interact with the C1q domain in *CTRP5*, suggesting that these two may be functionally related, similar to other dicistronic genes [13,25,26]. The *MFRP* gene in pigs is reported to be around 1,707 bp, encoding a putative protein of 508 amino acids, as found in the Ensembl browser [23]. Like human *MFRP*, pig *MFRP* also has 13 exons. Domain analysis of the p*MFRP* gene showed that it consists of two CUB domains (CUB1: 132 to 238 bp; CUB2: 289 to 403 bp), Ldla domain (244–287 bp) and a Frizzled domain (Fz: 454 to −563 bp). Unlike the human gene, only the monocistronic transcripts are reported for the pig *MFRP* and *CTRP5* genes in the Ensembl browser, suggesting that the bicistronic MFRP-CTRP5 transcripts may be absent in pigs.

Analysis of the p*CTRP5* DNA sequence noted a potential in-frame alternate start site upstream from the presumed coding sequence. Western blot expression analysis indicated that this potential in-frame start site was not translated (Figure 6). The 5′ RACE analysis of expressed p*CTRP5* mRNA from the RPE indicated that the TSS was located −65 bp to the presumed initiation codon and +77 bp from the potential in-frame alternate start site (Figure 2A). The TSS (−65 bp) of p*CTRP5* was similar in distance from the initiation codon, as we previously found with the independent hCTRP5 promoter (TSS −62 bp) [11]. Finally, the size of the pCTRP5 protein found in the pig eye was consistent with the predicted size (Figure 6B). Combined, these observations indicate that the cloned genomic pCTRP is expressed properly in vitro.

Apart from characterizing p*CTRP5* and pCTRP5, sequence analysis revealed the presence of an autonomous promoter for p*CTRP5*. We recently described an autonomous promoter function for h*CTRP5*; the results of the present finding are consistent with that report [11]. The sequences to the ATG start site in both human and pig *CTRP5* genes are highly conserved, with 73% homology. The cloned promoter region was capable of expression in both Cos-7 (Figure 5) cells and ARPE-19 cells (data not shown), indicating the functionality of the predicted promoter region. Further characterization of this promoter is necessary to determine the core promoter region.

CpG islands (CGIs) are clusters of CpG dinucleotides in GC-rich regions. They are also defined as regions with at least 200 bp, with a GC percentage that is greater than 50% and with an observed/expected CpG ratio that is greater than 60% [27]. In humans and mice, approximately 60% of all promoters colocalize with CpG islands [28]. Similar to h*CTRP5*, the CpG island search software predicted the presence of a CpG island in the 5′ region of the p*CTRP5* sequence. A single CpG island of 578 bp with a GC of less than 70.6% and an observed to expected CpG ratio of 0.66 was identified in p*CTRP5,* which may be involved in the initiation of DNA replication or transcription. The presence of a prominent CpG island and a conserved upstream region to the pCTRP5 gene confirms the presence of a regulatory region/promoter upstream to p*CTRP5*.

We have shown that the structure of the p*CTRP5* gene is similar to that found in humans [6,9]. The expression and localization of the pCTRP5 protein was also found to be consistent with the hCTRP5 expression in different cell lines, such as Cos-7 and ARPE-19 cells, indicating that these genes are highly similar not only in their structure but also in their function. These findings indicate that a pig model of LAZs and L-ORD is likely to mimic the human disease. The pig rod-to-cone ratio is around 8:1 for the total retina [17,29], compared to roughly 36:1 in the mouse retina [30]. The pig will thus be an excellent model for the human disease because it has a cone-rich retina and a macula-like region, the area centralis [16]. The pig has been used extensively as a model of retinitis pigmentosa [31] and is likely to serve as a useful model of LAZs and L-ORD.
